# Transesophageal echocardiography in orthotopic liver transplantation: a comprehensive intraoperative monitoring tool

**DOI:** 10.1186/s13089-017-0067-y

**Published:** 2017-06-19

**Authors:** Luigi Vetrugno, Federico Barbariol, Umberto Baccarani, Francesco Forfori, Giovanni Volpicelli, Giorgio Della Rocca

**Affiliations:** 1 Department of Medicine, University of Udine, P.le S. M. della Misericordia 15, 33100 Udine, Italy; 2 Anesthesia and Intensive Care Medicine IV, Pisa University Hospital, Pisa, Italy; 3 Department of Emergency Medicine, San Luigi Gonzaga University Hospital, Turin, Italy

**Keywords:** Transesophageal echocardiography, TEE, Liver transplantation, Caval anastomosis, Lung ultrasound

## Abstract

Intraoperative transesophageal echocardiography is a minimally invasive monitoring tool that can provide real-time visual information on ventricular function and hemodynamic volume status in patients undergoing liver transplantation. The American Association for the Study of Liver Diseases states that transesophageal echocardiography should be used in all liver transplant candidates in order to assess chamber sizes, hypertrophy, systolic and diastolic function, valvular function, and left ventricle outflow tract obstruction. However, intraoperative transesophageal echocardiography can be used to “visualize” other organs too; thanks to its proximity and access to multiple acoustic windows: liver, lung, spleen, and kidney. Although only limited scientific evidence exists promoting this comprehensive use, we describe the feasibility of TEE in the setting of liver transplantation: it is a highly valuable tool, not only as a cardiovascular monitoring, but also as a tool to evaluate lungs and pleural spaces, to assess hepatic vein blood flow and inferior vena cava anastomosis and patency, i.e., in cases of modified surgical techniques. The aim of this case series is to add our own experience of TEE as a comprehensive intraoperative monitoring tool in the field of orthotopic liver transplantation (and major liver resection) to the literature.

**Electronic supplementary material:**

The online version of this article (doi:10.1186/s13089-017-0067-y) contains supplementary material, which is available to authorized users.

## Introduction

The gold standard methods for monitoring cardiac output during liver transplantation surgery are pulmonary arterial catheterization and volumetric device monitoring (PiCCO/EV-1000); nonetheless experts agree that transesophageal echocardiography (TEE) is useful for managing rapid hemodynamic changes [1]. The American Association for the Study of Liver Diseases (AASLD) states that TEE should be used in all liver transplant candidates in order to assess chamber sizes, hypertrophy, systolic and diastolic function, valvular function, and left ventricle outflow tract obstruction [2].

The use of the TEE during liver transplantation to monitor cardiac function and to assess the inferior vena cava patency was described for the first time in 1992. In that case, the TEE probe was used in a patient with a suspected stricture of the suprahepatic inferior vena cava (IVC) anastomosis to evaluate its patency and the subsequent surgical intervention. The authors described how following separation from the venous–venous bypass, the liver appeared swollen, while the Doppler color flow imaging demonstrated marked turbulence in the suprahepatic IVC with narrow jet streaming into the right atrium [3]. With manual elevation of the liver, the pressure gradient disappeared, which allowed them to understand that the obstruction was due to the kinking of the vessel, and the anastomosis was revised. Color Doppler was once again performed and showed a decrease in the turbulence of the IVC blood flow. The case demonstrated that TEE can be used to assess the suprahepatic anastomosis, providing an early, non-invasive assessment of abnormalities that may require prompt correction. After this initial article, reports of TEE for the evaluation of hepatic vein structures have been scarce, despite the fact that TEE is becoming an increasingly popular tool in liver transplant surgery [4, 5].

## Study rationale and aim

**Fig. 1:**
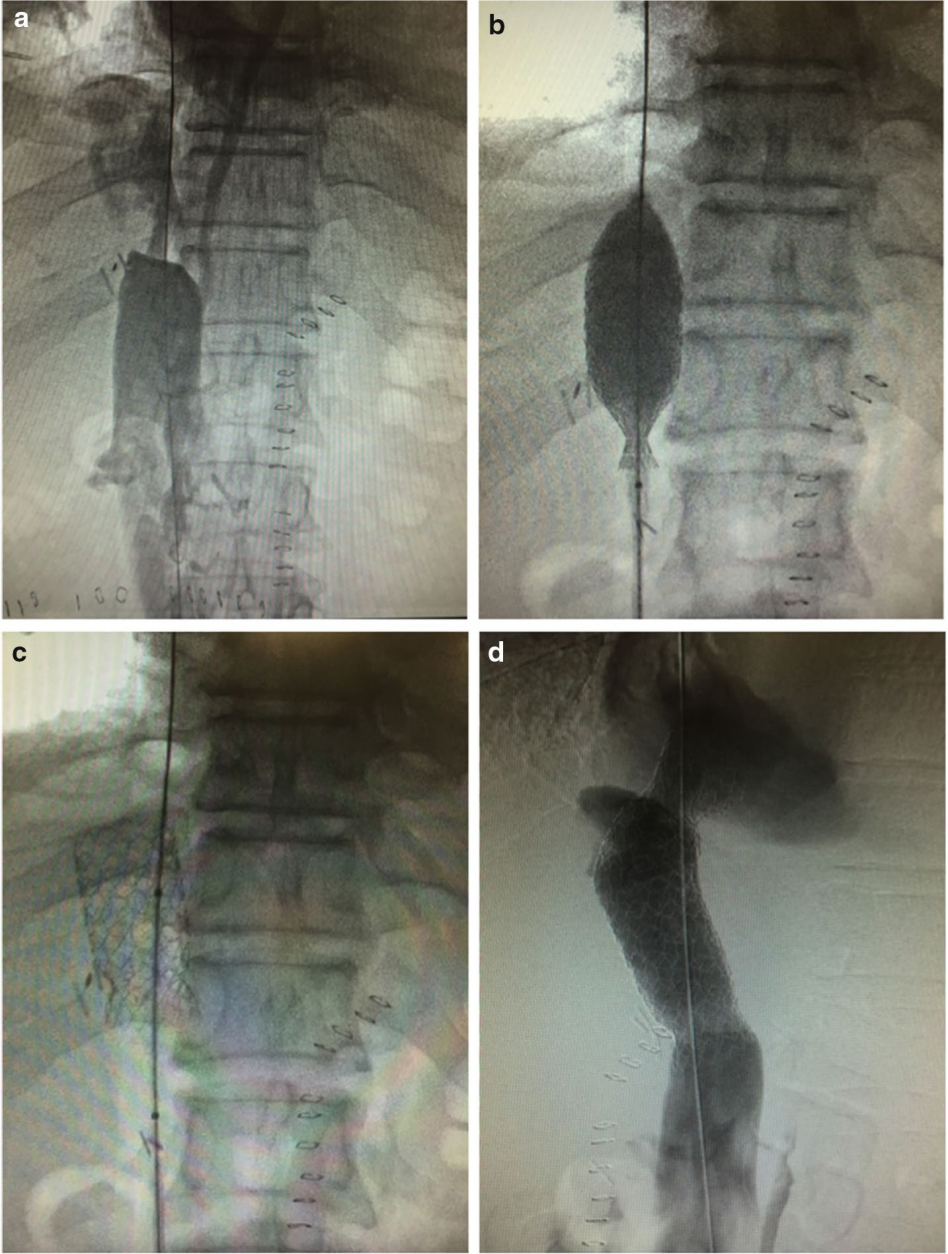
Percutaneous angioplasty and stent placement in a 63-year-old cirrhotic patient presenting suprahepatic IVC outflow compromise in the early postoperative period following orthotopic liver transplantation. Inferior vena cavogram demonstrates high-grade stenosis of the IVC (**a**); after vena cava angioplasty (**b**) and stent placement, a marked enlargement of IVC lumen is obtained (**c** and **d**)

Unrecognized IVC stenosis, thrombosis, and kinking during liver transplantation can lead to devastating consequences, requiring the patient to undergo further operations, such as surgical repair or re-transplant. The images in Fig. 1 show percutaneous transluminal angioplasty and double stent placement in a 63-year-old cirrhotic patient presenting suprahepatic IVC outflow compromise in the early postoperative period following orthotopic liver transplantation. The American Society of Echocardiography/Society of Cardiovascular Anesthesiologists now recommends that images of the IVC and hepatic veins are obtained as part of a comprehensive perioperative examination [6]. As reported in a recent editorial, although TEE has mainly been used in the context of the heart, it can also be used to “visualize” other organs. Inserting the probe beyond the bicaval view is helpful for assessing the IVC, the liver, and the hepatic veins. Furthermore, the literature contains many reports of TEE imaging of the pleura, the kidneys, and even the spinal cord [7–10].

The aim of this case series is to add our own experience of TEE as a comprehensive intraoperative monitoring tool in the field of orthotopic liver transplantation (and major liver resection) to the literature.

## Surgical considerations

The orthotopic liver transplantation technique was first described as a complete resection of the recipient’s IVC and interposition of the donor intrahepatic vena cava with two end-to-end anastomosis; for hemodynamic stabilization, the procedure also includes a veno-venous bypass, usually from left femoral to left axillary vein. In many centers, this approach still remains the gold standard [11]. In 1989 Tzakis et al. [12] introduced a new modified technique called the ‘piggy-back’ technique, which consists of preserving the full length of the recipient’s vena cava, but with anastomosis of the donor suprahepatic veins to the ostium of the recipient’s left and middle suprahepatic veins. The supporters of the piggy-back technique advocate the advantages of this procedure as the shorter operation times with a shorter anhepatic phase and warm ischemia, with one less anastomosis. This could reduce blood loss and give better hemodynamic stability without the use of a venous–venous bypass.

The ‘dark side’ of the piggy-back technique is the potential for venous outflow obstruction caused by size discrepancy between the donor suprahepatic vena cava and the recipient’s hepatic veins’ cuff: the associated risk of intra- or postoperative outflow problems of the graft has been reported to be between 1.5 and 8%, leading to major functional problems of the liver graft [13, 14]. For example, Cescon et al. experienced outflow problems in 4.6% of their patients, leading to a re-transplantation rate of 40%; outflow problems were also the cause of death in 23% of cases [15].

## Case 1

**Fig. 2:**
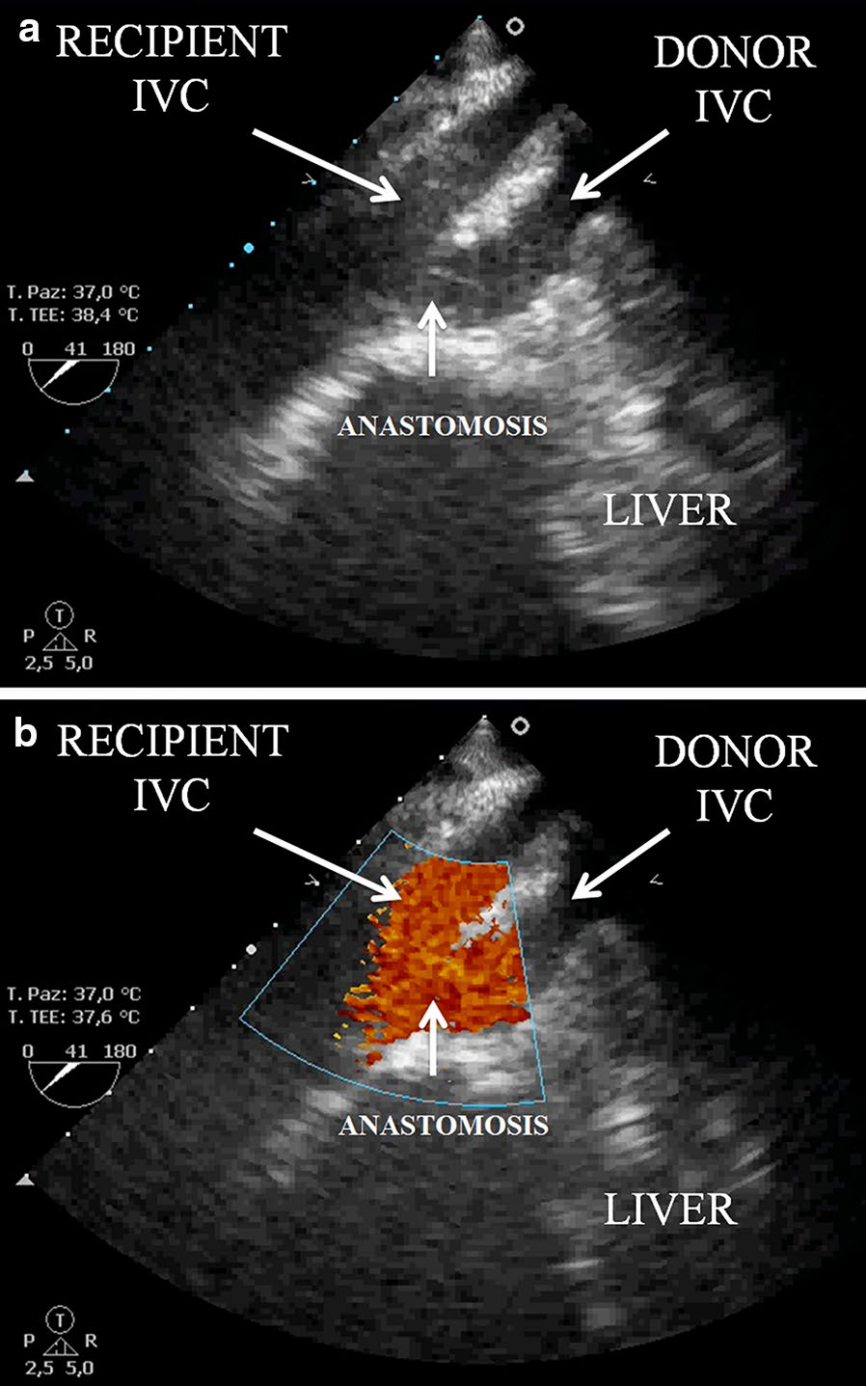
Transesophageal transgastric view at 41°, showing the double inferior vena cava in parallel manner without (**a**) and with (**b**) Doppler color flow through the patent terminolateral cavo-cavostomy

**Fig. 3:**
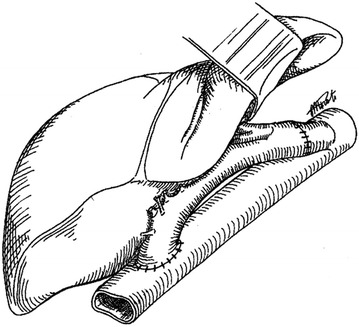
End-to-end infrahepatic rescue cavo-cavostomy (Reprinted from Transplant Proc. 2001; 33 (1–2):1389 with permission from Elsevier)

The images presented in Fig. 2 were acquired during liver transplant surgery in a 59-year-old patient with decompensated liver cirrhosis, severe portal hypertension, oesophageal varices (grade F2), and ascites secondary to previous alcohol abuse. After hepatectomy, performed using the piggy-back technique, [4] the donor suprahepatic IVC was anastomized to the hepatic vein cuff of the recipient, but the infrahepatic vena cava of the graft was oversewn. Following portal vein reperfusion, the graft started to swell and indurate in just a few minutes due to insufficient outflow. It is known that this condition, without correction, can lead to acute Budd–Chiari-like syndrome with graft loss in the immediate postoperative period [14]. The surgeons decided to perform a rescue terminolateral cavo-cavostomy by anastomosis of the donor infrahepatic vena cava to the recipient’s suprarenal vena cava to correct the inadequate outflow [16], as shown in Fig. 3.

**Fig. 4:**
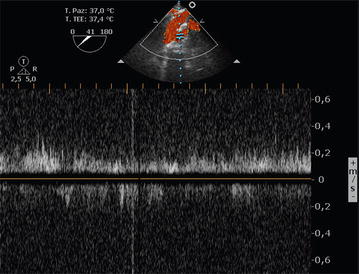
Doppler analysis of the blood flow through the cavo-cavostomy rescue anastomosis, showing appropriate liver venous drainage

A TEE examination of the liver was performed at the edge of the transesophageal transgastric view at 0 degrees; the probe was rotated clockwise and the phasic array signal emitted transducer moved between 40° and 60°, as previously described, to generate the basic hepatic view [17]. The donor and the recipient IVC were viewed in parallel, together with the flow through the cavocaval anastomosis, as reported in Additional file 1: Video 1, Additional file 2: Video 2, and Fig. 4. At the same time, the liver progressively assumed completely normal dimensions, color, and tissue consistency.

In the same patient, after the first declamping and before the cavo-cavostomy rescue anastomosis, a profound reduction in the preload due to the sequestration of blood in the liver and in the splanchnic circulation occurred, and adrenaline boluses were administered to support the hemodynamics. Because of this, the patient developed a transient ballooning syndrome that fortunately recovered after the increase in systolic pressure upon the second declamping following the cavo-cavostomy anastomosis (Additional file 3: Video 3).

## Case 2

**Fig. 5:**
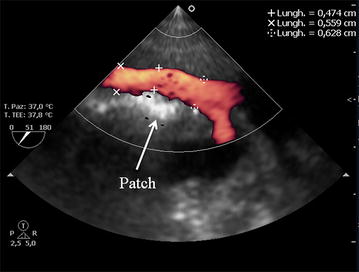
Color Power Angio Echography of the inferior vena cava blood flow through the patch repair. The Edwards^®^ Bovine Pericardial Patch appears as a hyper echoic structure in the anterior wall of the IVC (as shown by the *white arrow*). Diameters of the ICV before, through, and after the patch are shown

**Fig. 6:**
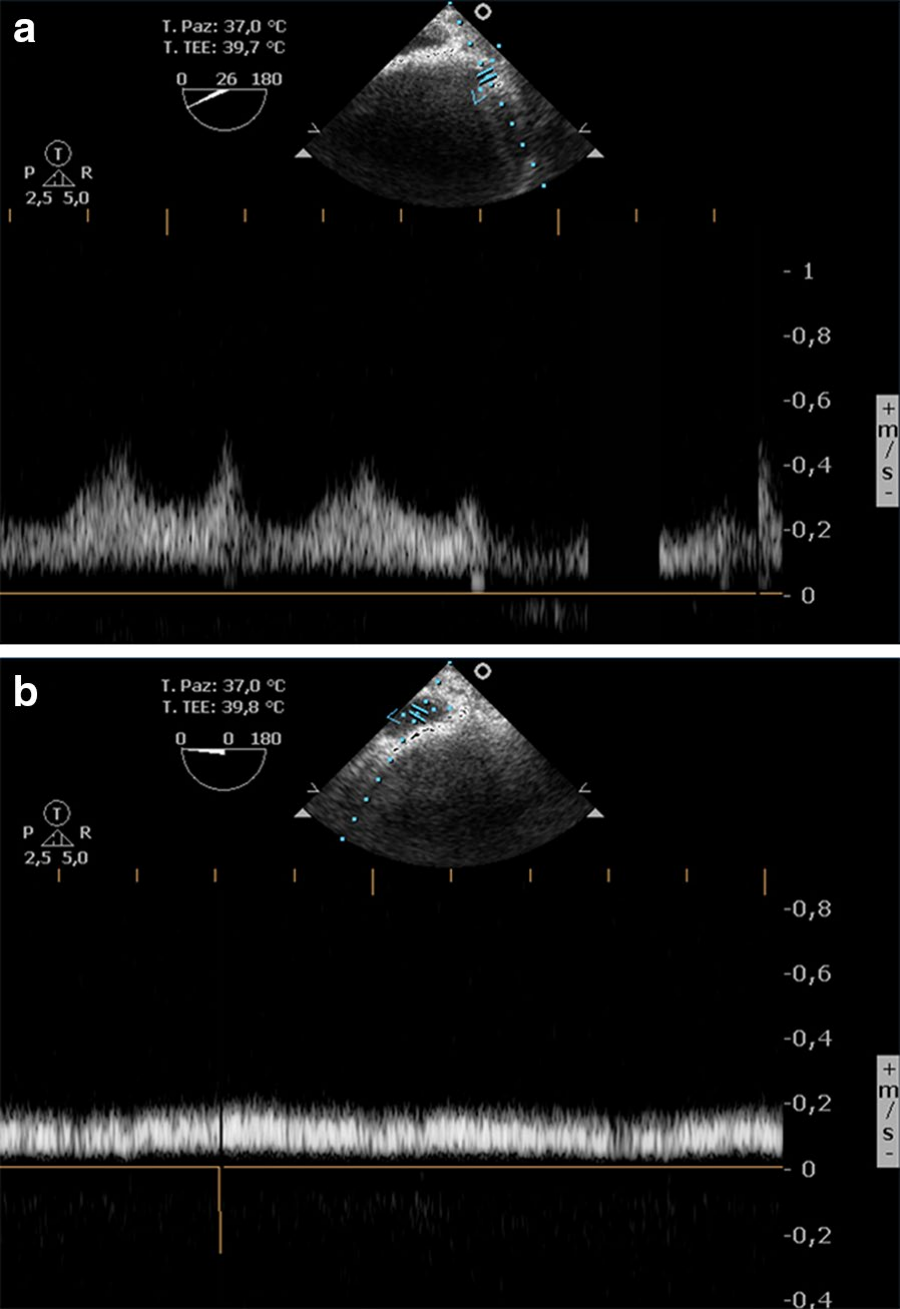
Pulse Wave Doppler (PW) analysis, measuring adequate blood flow velocity, in the suprahepatic vein (**a**) and inferior vena cava (**b**) after the patch repair

The images presented in Figs. 5, 6, and the related Additional file 4: Video 4 and Additional file 5: Video 5 were acquired after left lobe liver resection surgery in a 67-year-old woman with a giant hepatic angioma. During the surgical dissection of the tissues, an accidental laceration of the inferior vena cava occurred, which required almost complete clamping. To re-establish blood flow, the surgeons used an Edwards^®^ Bovine Pericardial Patch. After declamping, the surgeon requested that the patency of the inferior vena cava and blood flow in the suprahepatic veins were checked using TEE. A comparison of TEE for visualization of the main hepatic veins with the acquisition of Doppler ultrasound curves using the transabdominal approach was first described by Meierhenrich et al. [18] in their study of 34 patients scheduled for abdominal surgery. Using a multiplane TEE, they were able to identify the three main hepatic veins in all of the patients, and they reported that adequate Doppler tracings of the right and middle hepatic veins could be obtained in 100 and 97% of the patients, respectively, by TEE and in 91 and 50% of the patients by transabdominal sonography (TAS). Doppler tracing of the left hepatic vein could only be acquired in 18% of the patients by TEE, but in 47% of the patients by TAS. Doppler ultrasound and Pulse Wave Doppler (PW) analysis, which measure blood flow velocity (using a correct insonation angle, <60°), play an important role in the assessment of liver vessel patency, resistivity, and flow direction (as already shown in Figs. 5, 6, and Additional file 4: Video 4 and Additional file 5: Video 5).

**Fig. 7:**
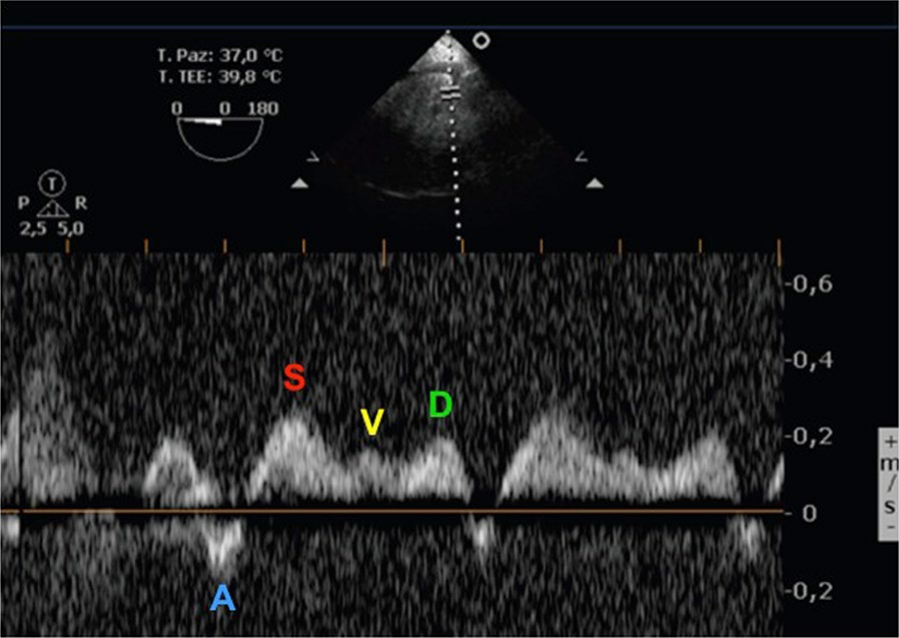
Normal triphasic hepatic Doppler waveform. The four waveforms constituting the normal spectral Doppler waveform are indicated: the A wave lies below the baseline (retrograde component); the S and D waves peak above the baseline and are caused by anterograde blood flow toward the right heart; and the V wave, a transitional wave, may peak just below or above the baseline

Usually, the normal hepatic vein waveform has four components: a retrograde A wave (right atrial contraction), an anterograde S wave (right ventricular systolic), a transitional V wave (pulmonary vein closure), and an anterograde D wave (right ventricular diastole)—see Fig. 7. The Doppler waveform of left hepatic vein blood flow resembles that of the pulmonary vein. It is important for the anesthesiologist to know that flow above the baseline indicates flow toward the transducer and flow below the baseline indicates flow away from the transducer. At least four questions need to be answered: Is there flow? Where it is directed (forward vs. backward)? What is the relationship of magnitude between the S and D waves (normal S>D vs. abnormal S<D)? What is the magnitude of the A wave (normal vs. abnormal)? A detailed explanation has been provided elsewhere [19].

## Case 3

During OLTX, the most dangerous phase regarding hemodynamic instability is the reperfusion phase, in which the surgical clamp is removed leading to a sudden release of cold, acidotic, oxidizing, and hyperkalaemic fluid into the circulation [20]. This may also be followed by reperfusion syndrome, occurring in about 42% of patients, which manifests as a temporary myocardial dysfunction with a reduction in heart rate, increased pulmonary arterial pressure, reduced cardiac venous return (preload), and a decrease in mean systemic blood pressure (afterload) of 30% [21]. In such cases, the re-stabilization of the heart becomes priority. In the case of reperfusion syndrome, the patient may experience low preload and afterload. If the patient has left ventricular hypertrophy and long anterior mitral leaflet, left ventricular outflow tract obstruction (LVOTO) may occur: the anterior leaflet of the mitral valve can occlude left ventricle outflow with severe posterolateral mitral regurgitation flow. Without prompt recognition and treatment, the patient could develop a profound cardiovascular collapse. It is important for the anaesthesiologist to be familiar with this possibility and its treatment, which principally consists of giving fluid to the patient, increasing afterload with vasoactive drugs, and reducing cardiac frequency. The images presented in Fig. 7 and the related Additional file 6: Video 6 were acquired in a 57-year-old HCV cirrhotic patient during the reperfusion phase of the liver transplant.

## Case 4

After induction of anesthesia in a 69-year-old patient undergoing liver transplantation, the patient suddenly developed desaturation, going from 100 to 90% of peripheral oxygen saturation in 50% oxygen ventilation. The patient did not have any history of hepato-pulmonary syndrome, and the preoperative chest X-ray was normal. Instead of performing recruitment maneuvers blindly, in the hope that the cause of hypoxia was due to the pressure of the diaphragm toward the lungs secondary to the abundant ascites, the TEE probe was inserted and the lungs explored, leading to a rapid diagnosis of right pleural effusion due to the passage of the ascites into the pleural space.

**Fig. 8:**
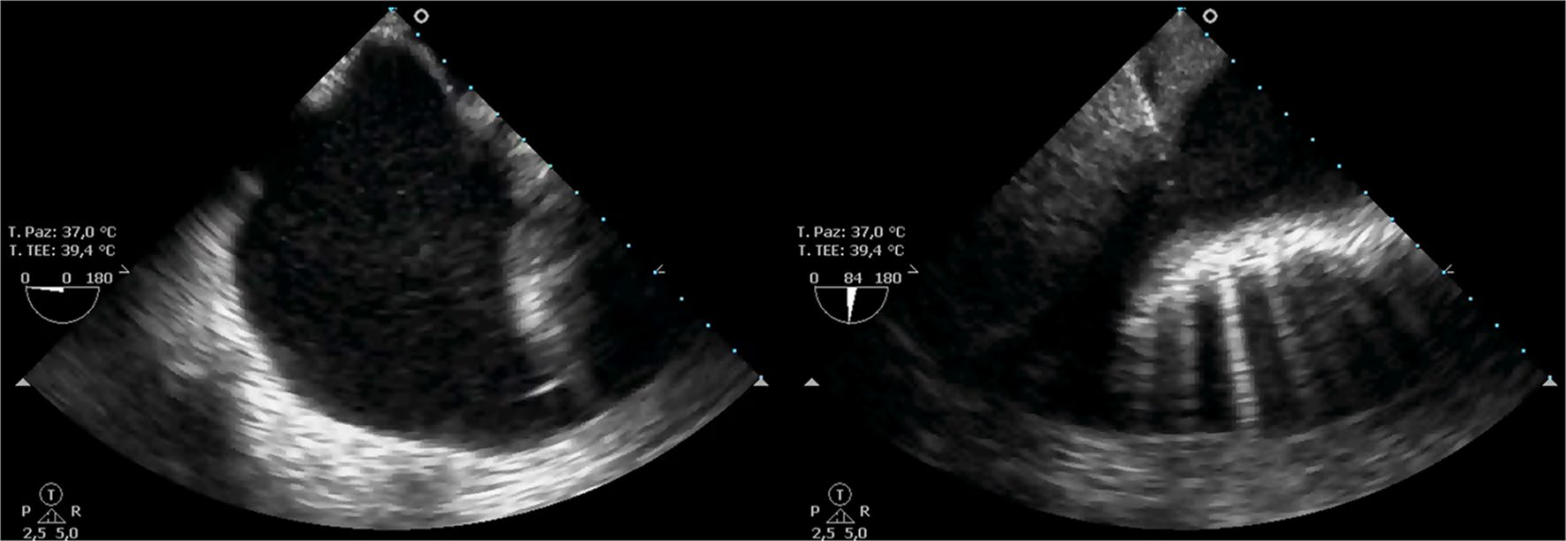
Pleural effusion in the chest cavity, as seen by trans-esophageal lung ultrasound (TELU) at 0° (*left image*) and 84° (*right image*). The effusion looks anechoic, while the lung parenchyma appears to be compressed, with some B-lines

The reported sensitivity of TEE for the detection of a pleural effusion is 97 with 100% specificity (compared with gold standard computed tomography) [22]. The patient underwent intraoperative pleural drainage and saturation was restored (see Fig. 8; Additional file 7: Video 7). As recently described by Cavayas et al. [23] transesophageal lung ultrasound (TELU) should be performed as a systematic approach. In TELU, the craniocaudal axis of each lung is divided into apical, middle, and basal regions; the left subclavian artery is used as a landmark to identify the apical regions; the superior pulmonary veins are used to mark the middle regions; and, finally, the inferior vena cava and the right atrial junction are used to identify the basal segments. From each of these landmarks, the lungs are scanned along the longitudinal axis by rotation of the ultrasound plane at 0° and 90°. In this case, the TEE probe was used because it was available as the point-of-care ultrasound methodology.

## Discussion

The goal of this paper was to describe how TEE in the setting of OLTX is a highly valuable (but maybe underutilized) tool, as it is important not only for cardiovascular monitoring, but also for evaluating hepatic vein blood flow and inferior vena cava anastomosis and patency. We describe, for the first time, the use of intraoperative TEE for evaluating the patency of a rescue cavo-cavostomy anastomosis in the intraoperative phase. TAS, on the other hand, is impossible to perform during surgery [16–18]. In fact, one of the major advantages of using a TEE probe is its anatomical proximity to the liver, allowing the liver to be studied from the inside without interfering with the surgery. Furthermore, TEE is more sensitive in detecting low blood flow velocity or small changes of blood flow in the hepatic veins [18]. Moreover, the literature reports a case in which intraoperative TEE evaluation of a vena cava tumor resection using the piggy-back technique led to a change in the surgical plain [24]. Thus, our report is not the first to demonstrate the feasibility of obtaining good liver anatomy visualization and acceptable quality images of the IVC through the use of TEE [25].

In patients undergoing liver transplantation, the occurrence of reperfusion syndrome can be dangerous, and visual assessment of the heart is of extreme importance. In the literature, very few reports describe dynamic left ventricular outflow tract obstruction, probably because TEE is used in just 50% of cases and because this condition is difficult to diagnose [26]. No reports describing the use of TEE in association with reperfusion syndrome or ballooning heart are present in the literature and the only case describing its use in liver transplantation is the one reported by Lee HR et al. [27] where it was used in the postoperative period. So our case is probably the first one.

Some limitations should be noted about the present study. First, the liver window is not always easy to obtain or adequate. Second, the presence of esophagus varices or gastric ulcers can limit the extensive use of TEE and its use must, of course, be balanced against the risks associated with performing the procedure [28]. As previously reported, patients with esophageal stricture, esophageal cancer, esophageal diverticulum, and recent esophageal surgery are generally considered to be near absolute contraindications for TEE [29]. Moreover, esophageal varices have been considered a relative contraindication to TEE, depending on the center and/or operator. Third, the anaesthesiologist should be skilled. However, TEE is safe and feasible in liver transplantation candidates. In fact, in a recent study, the major complication rate was just 0.86%, and the overall complication rate was 1.7%—both very low, but just slightly higher than that reported in cardiac literature in terms of morbidity rate of 0.2% [30].

## Conclusions

Echocardiography has become an invaluable tool in anesthesia practice and, thanks to its access to multiple acoustic windows, many organs (heart, liver, lung, spleen, kidney) can be explored in many perioperative diagnostic situations. During liver transplantation or major liver surgery, the use of TEE should not just be focused on the heart. It is our opinion that blood flow and patency of the hepatic anastomosis should be studied by TEE with the help of the radiologist before closing the abdomen. If this technique were to become adopted as standard procedure, then it would mirror what happened in cardiovascular surgery before cardiac anaesthesiologists became confident in using this tool.

## Additional files

**Additional file 1: Video 1.** The donor and the recipient IVC, viewed in parallel.

**Additional file 2: Video 2.** Color Doppler analysis of the blood flow through the cavo-cavostomy rescue anastomosis, showing appropriate liver venous drainage.

**Additional file 3: Video 3.** Different phases of the a transient ballooning syndrome developed after reperfusion, that recovered after the increase in systolic pressure upon the second de-clamping following a cavo-cavostomy anastomosis.

**Additional file 4: Video 4.** Color Doppler Echography of the inferior vena cava blood flow through the patch repair. The Edwards® Bovine Pericardial Patch appears as an hyper echoic structure in the anterior wall of the IVC.

**Additional file 5: Video 5.** Color Power Angio Echography of the inferior vena cava blood flow through the patch repair.

**Additional file 6: Video 6.** Left ventricular outflow-tract obstruction in a patient with left ventricular hypertrophy and long anterior mitral leaflet: the anterior leaflet of the mitral valve occludes left ventricle outflow with severe posterolateral mitral regurgitation flow.

**Additional file 7: Video 7.** Pleural effusion in the chest cavity, as seen by trans-esophageal lung ultrasound (TELU) at 0° and subsequently 84°. The effusion looks anechoic, while the lung parenchyma appears to be compressed, with some B-lines.

## Abbreviations

IVC: inferior vena cava
TELU: transesophageal lung ultrasound
TTE: transesophageal echocardiography
